# Current News

**Published:** 1903-06

**Authors:** 


### Current News.

### PENNSYLVANIA STATE DENTAL SOCIETY—SPECIAL NOTICE.

Owing to the number of inquiries and demand for accommodations the Committee have been compelled to secure larger quarters for the meeting of the Pennsylvania State Dental Society, to be held July 7, 8, and 9, 1903, and have decided on Hotel Sterling, in Wilkes-Barre, Pa.

H. B. McFadden, D.D.S.,
Howard S. Seip, D.D.S.,
James P. Nicol, D.D.S.,
*Executive Committee.*

May 1, 1903.

### NEW JERSEY STATE DENTAL SOCIETY.

The thirty-third annual meeting will be held at the Auditorium, Asbury Park, commencing at ten a.m., Wednesday, July 15, and continuing the 16th and 17th. Four papers will be read by eminent members of the profession, and, as with last year's new departure, the clinics will be made a great feature of the meeting. Forty clinicians have promised to be present, and the immense floor-space at the disposal of the society will be filled with everything new and novel in dentistry.

The Columbia Hotel opposite the Auditorium will be the headquarters, and the rates $2.50 and $3.00 per day. Mark off the date to-day, and come July 15. You will be made welcome.

Charles A. Meeker,
*Secretary.*

### NATIONAL DENTAL ASSOCIATION.

The next annual session of the National Dental Association will be held in Asheville, N. C., commencing Tuesday, July 28, 1903. Preparations are being made for one of the best meetings in the history of the Association. The Section officers are preparing a programme which, from a scientific and practical stand-point, will be difficult to excel. The clinics will be made a special feature.

All dentists interested in the advancement of the profession should attend this meeting.

All State and local societies should elect delegates who will be sure to attend the National meeting, they being entitled to one delegate for every six of their members.

The usual railroad rates will be had on all roads in the United States and part of Canada,—one fare and a third, on the certificate plan.

L. G. NOEL, *President.*
A. H. PECK, *Recording Secretary.*

### NATIONAL ASSOCIATION OF DENTAL FACULTIES.

THE National Association of Dental Faculties will convene in the Ball-room of the Battery Park Hotel, Asheville, N. C., Friday, July 24, at eleven A.M.

The Executive Committee will meet at same place Thursday, July 23, at 2.30 P.M. All parties having special business with this committee are hereby notified to be on hand at this time.

H. B. TILESTON, *Chairman,*
S. W. FOSTER, *Secretary,*
*Executive Committee N. A. D. F.*

### HARVARD ODONTOLOGICAL SOCIETY.

THE Harvard Odontological Society held its twenty-fifth annual meeting and ladies' night on the evening of February 28, 1903, at Young's Hotel, Boston.

President Julius G. W. Werner being absent because of sickness, Recording Secretary John W. Estabrooks presided.

There were seventy-one guests and members present.

After a reception and banquet the society and guests were entertained by an illustrated lecture entitled "Argentina," by Mr. George H. Worthley, of Brookline, Mass. Music was furnished by W. S. Kerr, soloist, and Mrs. William B. Eddy, pianist.

This society enters upon its twenty-sixth year under the most promising conditions, there being eighty-nine active, fifteen corresponding, and two honorary members.

The officers elected for the ensuing year are, President, Julius G. W. Werner, D.M.D.; Recording Secretary, John W. Estabrooks, D.M.D.; Corresponding Secretary, Arthur H. Stoddard, D.M.D.; Treasurer, H. Winchester Hardy, D.M.D.; Editor, Horace L. Howe, D.M.D.

*Executive Committee.*—John W. Estabrooks, D.M.D., William P. Cooke, D.M.D., Lyman F. Bigelow, D.M.D.

ARTHUR H. STODDARD,
*Corresponding Secretary.*

### SOUTH DAKOTA DENTAL SOCIETY.

THE annual meeting of the South Dakota Dental Society will be held at Redfield, S. D., June 3, 4, and 5, 1903. A fine programme is assured, in charge of Dr. J. P. Buckley, of Chicago. All reputable dentists of this and other States cordially invited. The State Board of Dental Examiners will be in session at the same time and place.

D. ST. I. DAVIES, *President,*
Woonsocket, S. D.
W. W. PRICE, *Secretary,*
Centerville, S. D.

### WISCONSIN STATE DENTAL SOCIETY.

THE thirty-third annual meeting of the Wisconsin State Dental Society will be held at West Superior, Wis., July 21, 22, and 23, 1903. The usual railroad rates will be obtained. The profession is cordially invited to be present.

T. M. WELCH, *President.*
W. H. MUELLER, *Secretary.*

MADISON, WIS.

